# Utilization of Solid Waste from Brick Industry and Hydrated Lime in Self-Compacting Cement Pastes

**DOI:** 10.3390/ma14051109

**Published:** 2021-02-27

**Authors:** Mati Ullah Shah, Muhammad Usman, Muhammad Usman Hanif, Iqra Naseem, Sara Farooq

**Affiliations:** School of Civil and Environmental Engineering, National University of Sciences and Technology (NUST), H-12 Sector, Islamabad 46000, Pakistan; shahsmathishah@gmail.com (M.U.S.); Usman.hanif@nice.nust.edu.pk (M.U.H.); iqra.naseem@nice.nust.edu.pk (I.N.); sarafarooq.hu@yahoo.com (S.F.)

**Keywords:** cement paste, supplementary cementitious materials (SCMs), waste burnt brick powder (WBBP), self-compacting paste systems (SCPs), shrinkage, rheological properties, durability, hydrated lime (HL)

## Abstract

The huge amount of solid waste from the brick manufacturing industry can be used as a cement replacement. However, replacement exceeding 10% causes a reduction in strength due to the slowing of the pozzolanic reaction. Therefore, in this study, the pozzolanic potential of brick waste is enhanced using ultrafine brick powder with hydrated lime (HL). A total of six self-compacting paste mixes were studied. HL 2.5% by weight of binder was added in two formulations: 10% and 20% of waste burnt brick powder (WBBP), to activate the pozzolanic reaction. An increase in the water demand and setting time was observed by increasing the replacement percentage of WBBP. It was found that the mechanical properties of mixes containing 5% and 10% WBBP performed better than the control mix, while the mechanical properties of the mixes containing 20% WBBP were found to be almost equal to the control mix at 90 days. The addition of HL enhanced the early-age strength. Furthermore, WBBP formulations endorsed improvements in both durability and rheological properties, complemented by reduced early-age shrinkage. Overall, it was found that brick waste in ultrafine size has a very high degree of pozzolanic potential and can be effectively utilized as a supplementary cementitious material.

## 1. Introduction

Self-compacting concrete (SCC) is an unconventional and revolutionary concrete technology. It can flow under its own weight without bleeding and segregation and can achieve full compaction without any mechanical effort [1,2]. These unique properties of SCC are attributed to the high volume of self-compacting paste (SCP) in SCC [3]. SCP acts as a lubricant to aggregates in SCC [4]. Consequently, SCP plays a vital role in controlling the properties of SCC. A huge amount of cement is required in SCP that not only increases the cost of SCC as compared to conventional concrete but also affects the concrete durability [5]. Apart from cost and durability-related problems, the high consumption of cement adds to environmental pollution. It has been reported that one ton of clinker production emits 850 kg of CO_2_ [6,7]. To make SCC economical as well as durable, many researchers have recommended mineral admixtures such as fly ash [8], silica fumes [9], blast furnace slag [10,11], rice husk ash [12,13], bentonite [14], limestone powder [15], waste clay products [16], and copper slag [17] to be used as cement replacement. When mineral admixtures are added to the concrete as a cement replacement, they either act as a pozzolanic agent or an inert filler material to change the properties of the concrete depending on the nature of the mineral admixture. SCP properties are not only governed by the chemical properties of supplementary cementitious material (SCM) but also the physical properties such as particle shape, size, and morphology. Such properties play an important role in defining the performance of SCP [18]. Therefore, a detailed understanding of SCM is necessary before using it as a mineral admixture in concrete.

Various researchers suggested that the construction industry can become sustainable by using industrial and construction waste as a building material [10,19]. Using this waste will not only reduce the cost of concrete but will also have considerable positive environmental impacts [20,21,22]. Waste from timber [23,24], coal plants [25], marble [26], rubber [27], ceramic [28,29,30], and brick industries [31,32,33,34] has great potential to contribute to sustainable construction. Yearly, construction and demolition waste (C&DW) is generated around 3 billion tons worldwide [35]. The major C&DW in developing countries such as China, India, and Pakistan is concrete and bricks. Around 1.5 trillion bricks are manufactured each year worldwide. Out of that 1.5 trillion, 1.3 trillion bricks are produced in Asia. Brick production per annum in China is 1 trillion, and in India, it is almost 250 billion [36]. According to the National Action and Coordination Group (NACG), Pakistan is the third-largest brick-producing country in Asia, where more than 45 billion bricks are produced every year [37]. A huge quantity of solid waste is generated during the production of bricks, at the construction site, and from the demolition of old existing masonry structures. Unfortunately, these developing countries do not have an efficient system to monitor and use C&DW as construction materials. Therefore, this C&DW occupies acres of areas of landfills. Before it becomes too late, there is an immense need at the government level in such developing countries to formulate a system for the effective utilization of C&DW. The C&DW of bricks can be reused as an aggregate or as a secondary raw material as a cement replacement in concrete.

It has been reported that waste burnt brick (WBB) in fine powder form has pozzolanic properties and can be used as SCM to enhance the properties of concrete [38,39,40,41,42]. Abib et al. reported that a mix containing 5% of waste burnt brick powder (WBBP) as a replacement showed superior mechanical and rheological properties than the control formulation [42]. Naceri and Hamina reported that a 10% replacement of cement with WBBP has similar mechanical properties to the control sample at 90 days [41]. A small replacement percentage of WBBP with cement in concrete has a minor strength loss at the early ages and exhibits very good pozzolanic reactivity [39]. However, the strength of concrete is reduced when the cement replacement with WBBP is greater than 10% [40], especially at the early ages in such cases where the strength is very low [41]. There are many reasons for the reduction in strength in mixes having a higher replacement percentage of WBBP. One of the reasons is the coarser particle size of WBBP. If the grain size of WBBP is coarser, then it will act as an inert filler rather than a pozzolan [43]. Consequently, strength reduction will occur at higher replacement. Another cause for the reduction in strength is that enough portlandite for a pozzolanic reaction is not available [44] at the early ages. 

Pozzolanic materials may or may not be cementitious themselves, but in a finely divided form and in the presence of moisture, they react with the cement hydration product portlandite to give cementitious properties. In case of a small percentage replacement of cement, sufficient portlandite is available. Therefore, SCM quickly starts to contribute to strength through pozzolanic activity [45]. However, mixes at a high replacement percentage of cement by SCM do not have sufficient portlandite to contribute fully through pozzolanic activity [46,47]. Therefore, a large part of the pozzolan acts only as a filler. As a consequence, concrete containing pozzolanic materials has low strength at the early ages [46,47]. Many practical solutions have been presented to increase the rate of the pozzolanic reaction in SCM formulations at the early stages of hydration, such as fine grinding [43,48] and high curing temperatures [46]. Both methods can enhance the strength in SCM formulations to an equivalent value of the control Portland cement at the early ages [45]. However, these solutions are not economical. Alternate options can be alkaline activators [49,50] or sulfate-based activators [50]. The application of such activation mechanisms can, however, trigger durability issues. Alkaline activation can lead to an alkali–silica aggregate reaction, and sulfate-based activation can affect the durability of concrete due to the formation of a large amount of ettringite [51]. Apart from alkaline and sulfate-based activation mechanisms, different studies suggest that the initial strength of concrete recipes containing pozzolanic materials can be improved with a small addition of silica fume, hydrated lime (HL), or limestone fines [46,51,52]. 

The application of waste burnt bricks as a cement replacement has been studied by many researchers [31,40]. WBBP can be used in higher ratios, i.e., greater than 10%, to address economical and sustainability issues, but its inefficiency to perform as a pozzolanic material has not been addressed until now [40]. In the current study, a unique and novel approach was used to improve the pozzolanic behavior of a WBBP replacement higher than 10%. That approach is using ultrafine particles of WBBP with HL as a pozzolanic activation agent. HL, as an activation agent with ultrafine particles of SCM, has not been studied before. Keeping the uniqueness of this work in view, three well-defined objectives were devised. The first objective was to improve the pozzolanic potential of WBBP using HL with ultrafine particles of WBBP. The second objective was to evaluate the rheological and mechanical properties of SCP formulations with and without WBBP and HL. The third objective was to assess the durability of SCP formulations and evaluate the microstructure of the paste with and without WBBP and HL. Cement was replaced by WBBP at various percentages ranging from 5% to 20%. The pozzolanic potential of WBB is made effective in two ways. First, the degree of the pozzolanic potential of WBB is increased when using it in ultrafine powder form (median size of a particle is 2.76 µm) in all six formulations. Second, HL is added to 10% and 20% replacement mixes to improve the pozzolanic reactivity of WBB. Material characterization is performed using scanning electron microscopy images (SEM) and X-ray diffraction (XRD). Fresh state properties are discussed in detail, especially concerning the water and superplasticizer (SP) demand, early-age shrinkage, flow time, etc. The hardened state samples are also analyzed using SEM and XRD in addition to mechanical properties. Moreover, the durability of the self-compacting paste subject to acid attack is also studied. Many interesting aspects are discussed, highlighting several benefits of the proposed sustainable self-compacting pastes.

## 2. Methodology

### 2.1. Mix Proportion of SCP Formulation

A total of six formulations were studied at their respective Vicat water demand (WD) and superplasticizer (SP) demand. Out of six formulations, three were prepared by incorporating WBBP as SCM at 5%, 10%, and 20% by weight of cement and in two formulations besides WBBP, 2.5% by weight of binder (cement + WBBP) HL was used as a chemical modifier. The water/cement ratio (w/c) was added based on the normal consistency of each formulation. Superplasticizer (SP) was added as a percentage of cement. The details of the formulations are shown in Table 1. The typical formulations in Table 1 such as C90-HL can be read as 90% cement, w/c = 31%, SP = 0.37% of cement, 10% WBBP, and HL at 2.5% of binder.

### 2.2. Material Characterization

#### 2.2.1. Cement

**Figure 1:**
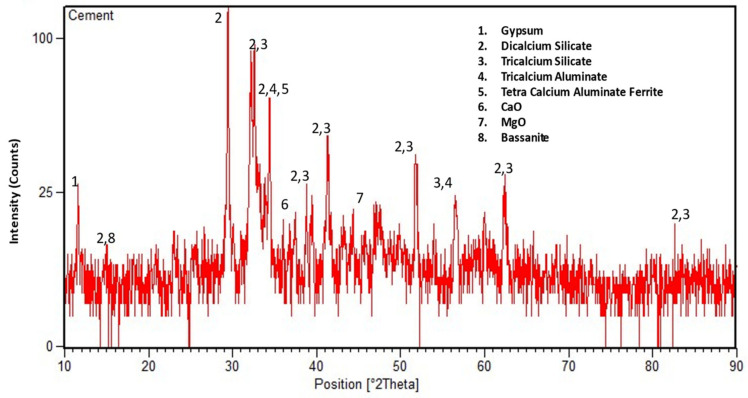
X-ray diffraction of ordinary Portland cement (OPC) Type-I.

**Figure 2:**
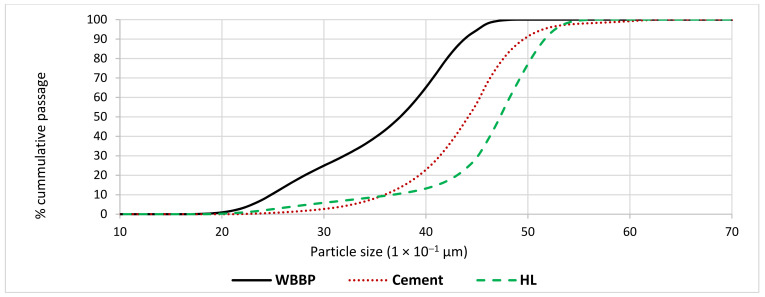
The particle size distribution of cement, waste burnt brick powder (WBBP), and hydrated lime (HL).

Locally available Type-I ordinary Portland cement (OPC) conforming to ASTM C-150 was used during the testing [53]. The X-ray diffraction (XRD) analyzer was used to find the mineral composition of OPC. Copper (Cu) was selected as an X-ray target, and the diffractograms were recorded at a wavelength of 1.54 Å with typical peaks attained at different 2-theta, as shown in Figure 1. Dominant mineral phases of OPC such as tricalcium silicate, dicalcium silicate, tricalcium aluminate, and tetra calcium aluminate ferrite were detected in XRD. Other chemical compounds of OPC such as gypsum, CaO, and MgO were also observed in the XRD profile. The particle size distribution (PSD) of cement was determined using a laser particle size analyzer. The PSD curve (Figure 2) shows that cement grains are coarser than WBBP particles and finer than hydrated lime particles. The chemical oxide composition was determined through the X-ray fluorescence spectrometer, as shown in Table 2. The physical properties of OPC are displayed in Table 3.

#### 2.2.2. Burnt Brick Powder

**Figure 3:**
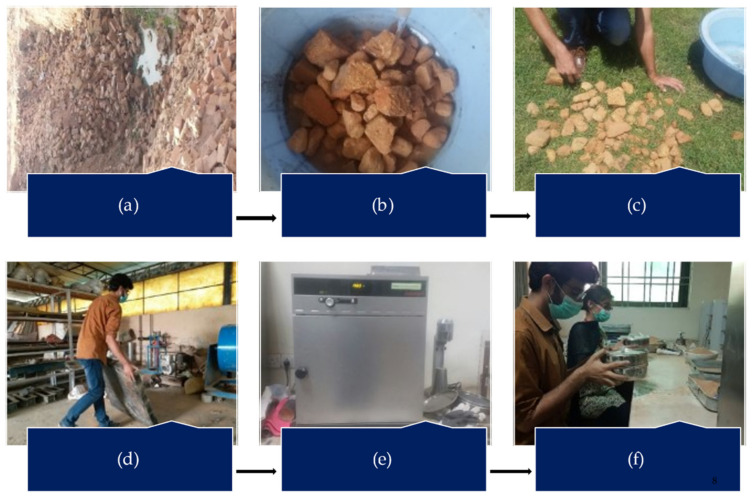
Preparation process of waste burnt brick powder (WBBP): (**a**) collection site of waste bricks; (**b**) collected bricks are crushed into smaller size and washed to remove dirt; (**c**) the wet pieces of waste bricks are dried under sunlight for 1 day; (**d**) The sun-dried sample is converted into powder using the ball milling process; (**e**) to remove any moisture content, the WBBP is oven-dried for 24 h; (**f**) the oven-dried powder is converted into ultrafine particles through manual pulverization, and then it is sieved through a 75 µm sieve and stored in airtight buckets.

**Figure 4:**
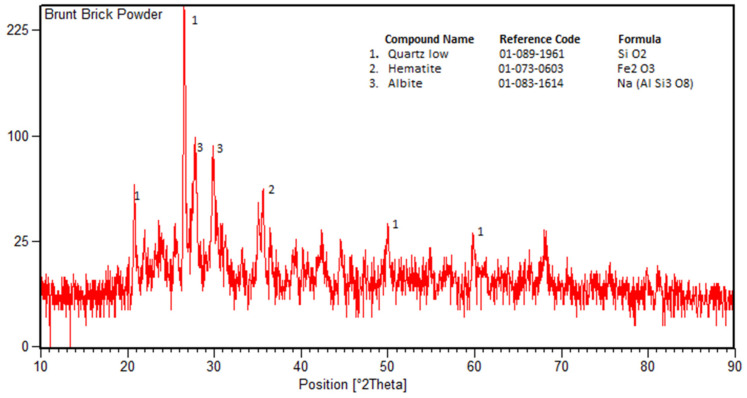
X-ray diffraction of waste burnt brick powder (WBBP).

**Figure 5:**
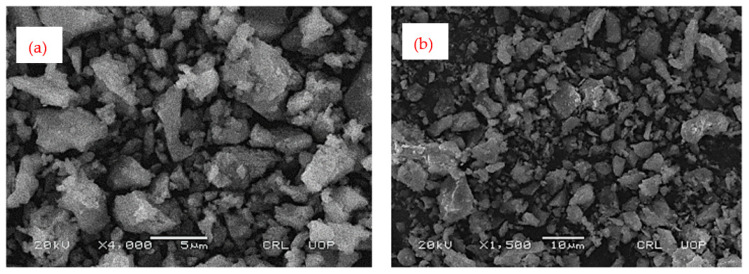
Scanning electron microscopy images of waste burnt brick powder: (**a**) at 5 µm resolution; (**b**) at 10 µm resolution.

The solid waste burnt brick (SWBB) powder used in this study was collected from a local source at Nonagran Kiln situated in the vicinity of Islamabad, Pakistan. The obtained SWBB was washed first and then dried in sunlight for one day. After the sun drying process, waste bricks were ground in a ball milling machine and then oven-dried for 24 h. The oven-dried finely divided WBBP was passed through the #200 sieve (75 µm) and stored in airtight buckets (Figure 3). The XRD pattern of WBBP (Figure 4) indicates the presence of quartz, hematite, and albite minerals. The free silica quartz in WBBP comes from its sand and clay component, while the amorphous aluminosilicate comes from the decomposition of clay minerals, hematite, and albite [54]. It can be seen in the PSD (Figure 2) that WBBP particles (median size 2.76 µm) are much finer than OPC (median size 6.85 µm) and hydrated lime (HL) (median size 10.47 µm). The chemical oxide composition and physical properties of WBBP are listed in Table 2 and Table 3, respectively. The physical, as well as chemical, properties of WBBP meet the ASTM C-618 requirements of pozzolanic materials [55]. Scanning electron microscopy images (SEM) of WBBP at different resolutions in Figure 5a,b show that particles of WBBP are very fine, irregular in shape, rough in texture, and have a porous surface.

#### 2.2.3. Hydrated Lime

Hydrated lime was purchased from a local source in Taxila, Pakistan. HL is coarser than WBBP and OPC (Figure 2). The chemical properties and physical properties are tabulated in Table 2 and Table 3, respectively.

#### 2.2.4. Water

Potable drinking water at the temperature of 25 ± 1 °C was used for mixing. The properties of the water are shown in Table 4. The water properties meet the World Health Organization (WHO) guidelines for drinking water. Therefore, no adverse effects on the properties of the cement paste occurred due to the water.

#### 2.2.5. Admixture

Viscocrete, a third-generation 3110 superplasticizer which is an aqueous solution of the modified poly-carboxylate copolymer, was used in the current study. It was present in liquid form and conformed to the ASTM C494-86 type GF requirement [56]. The physical properties are presented in Table 5.

### 2.3. Mixing Regime, Casting, and Curing

All mixings were conducted using a 5 L capacity Hobart Mixer. The mixing regime conforming to the ASTM C-305-14 [57] standard was adopted for all tests to ensure uniformity in sample preparation. All the dry ingredients of the paste were first manually mixed in a closed container to ensure the uniformity of the batch. The uniformly mixed powder was then fed into the bowl of the mixer containing the required mixing water with the liquid admixture and allowed to be absorbed in the water for 30 s. Slow mixing (140 ± 5 r/min) was conducted for 30 s and then the mixer was stopped for 15 s to scrape any paste material that adhered to the sides of the bowl. Thereafter, the batch was mixed for 60 s at (285 ± 10 r/min). The prisms of size 40 mm × 40 mm × 160 mm were cast immediately from the paste after mixing and left for 24 h in the casting room at a temperature of 20 ± 1 °C and relative humidity of 100%. Then, the specimens were de-molded and immersed in a 1000 L curing tank at a temperature of 23 ± 2 °C. The used curing facility meets the requirements of ASTM C-511 [58]. The water of the curing tank was lime-saturated to avoid the leaching out of calcium hydroxide from test specimens. Specimens were cured as per ASTM C-192 [56] until the time of testing. On the day of testing, specimens were taken out from the curing tank and kept for air drying at least 1 h before testing. A total of 174 prisms and 54 (50 mm) cubes were cast for different tests.

### 2.4. Experimental Program

#### 2.4.1. Fresh Properties

##### Water and Superplasticizer Demand

Water demand (WD) is the minimum amount of water required for the standard consistency of cement paste. WD was determined conforming to the procedure of ASTM C-187-16 [59] using a Vicat apparatus manufactured by Controls, Milano, Italy, for each formulation. The water demand of each formulation was taken as an average of three readings. The WDs of C90-HL and C80-HL were kept the same as those of C90 and C80, respectively. Superplasticizer (SP) demand was determined using Hagerman’s mini-cone dimensions, 6 cm × 7 cm × 10 cm. SP dosage was determined through a trial-and-error procedure until the average target flow of 30 ± 1 was achieved [54]. The SP demand of each formulation was based on an average of three readings.

##### Flow and Setting Time

Flow time is an important parameter that provides a general idea about the characteristics of fresh paste. The purpose of a flow test using Hagerman’s mini-slump cone is to investigate the yield stress, deformability, and unrestricted filling ability of SCP systems. Therefore, the flow test measures two parameters: total flow spread and flow time. T25 cm and T30 cm are the times the paste takes to reach 25 and 30 cm after the paste leaves the Hagerman mini-slump cone. Rizwan reported that the flow time gave an indirect measure of the viscosity and yield strength of the SCP [60]. T25 cm indicates yield strength and T30 indicates the rate of deformation or viscosity of SCP within a defined flow distance. The smaller the flow time, the less internal friction offered by powder particles, so the higher time will be the deformation. The ASTM C 191-19 procedure was adopted to determine the setting time of SCP mixes at their respective WD and SP demands using the Vicat apparatus [61].

##### Early-Age Shrinkage

Shrinkage is the reduction in the volume of cement paste after the start of the hydration process. The reduction in the volume of the cement paste can be a result of several parallel operating shrinkage mechanisms including autogenous shrinkage, drying shrinkage due to loss of water to the surroundings, and shrinkage due to carbonation [62]. In the current study, the “Modified German Schwindrine” apparatus measuring 4 cm × 6 cm × 25 cm, interfaced with a computer, and having a sensitivity of 0.31 microns/m was used to measure the linear early shrinkage response of SCP formulations at 20 ± 1 °C for 24 h. The samples were not sealed during the experiment. Further, the isothermal condition for shrinkage measurement with the apparatus was not possible. Therefore, it measured the total shrinkage of the cement paste. The data recording of shrinkage started after 5 min of paste mixing and recorded for 24 h.

#### 2.4.2. Hardened State Properties

##### Mechanical Strength

The flexural and compressive strengths of SCP formulations were determined as per ASTM C-348 [63] and ASTM C-349 [64], respectively. The flexural strength at any age is an average of three samples (40 mm × 40 mm × 160 mm), while the compressive strength is found as an average of six samples obtained from broken pieces of the flexural test with a cross-section of 40 mm × 40 mm.

##### Water Absorption

Water absorption was determined according to ASTM C642-97 specifications [65] on prisms of 40 mm × 40 mm × 160 mm. After 28 days of curing, the samples were placed in an oven at 100° C for 24 h. After 24 h, oven-dried samples were weighed and then immersed in water for 48 h, after which samples were removed and weighed in the saturated surface dry (SSD) condition. Water absorption was thus calculated from the difference between the oven-dried and SSD weights. The waster absorption of each formulation was based on an average of two readings.

##### Resistance against Acid Attack

Concrete containing SCM is more durable against the acid attack. The durability of concrete against acid attack is usually measured in terms of the percentage reduction in weight and strength loss when exposed to the acidic environment. The reduction in the weight of paste when exposed to the acidic environment is due to the loss of cohesiveness of the cement hydration products [66]. The hydration product such as calcium hydroxide (CH) reacts with the acid to precipitate gypsum [67]. Precipitation of gypsum causes expansion and cracking in the cement matrix. Once CH is depleted, the pH of the pore solution decreases, and as a result, the decalcification of the calcium silica hydrate (C–S–H) phase initiates causing the increase in porosity and degradation of the cement paste [68,69,70,71].

For durability assessment of SCP formulations, nine (50 mm) cubes of each formulation were cast and cured for 28 days. After curing, the samples were dried and weighed. Three samples of each formulation were left in open air. Meanwhile, out of the remaining six specimens of each formulation, half were immersed in 2.5% (percentage by weight) H_2_SO_4_ solution (8 L) and the other half in 2.5% (percentage by weight) HCL solution (8 L) for 30 days. Both the solutions were renewed after 15 days. At the age of around 60 days, the samples were removed from the acidic solution. After that, specimens were left to dry, and their weight reduction was noted. Then, nine specimens of each formulation were tested in compression conforming to ASTM C-109 [72], and a reduction in strength was found.

#### 2.4.3. Microstructure Investigation

For detailed microstructure investigation, scanning electron microscopy (SEM) and X-ray diffraction (XRD) were conducted. The useful information regarding the changes that occurred in the microstructure and morphology of the hydration products of SCP systems through WBBP and HL was extracted from micrographs using SEM. Different hydration products were identified through XRD in some selected formulations of SCP with and without WBBP.

## 3. Results and Discussion

### 3.1. Fresh Properties

#### 3.1.1. Water Demand (WD)

**Figure 6:**
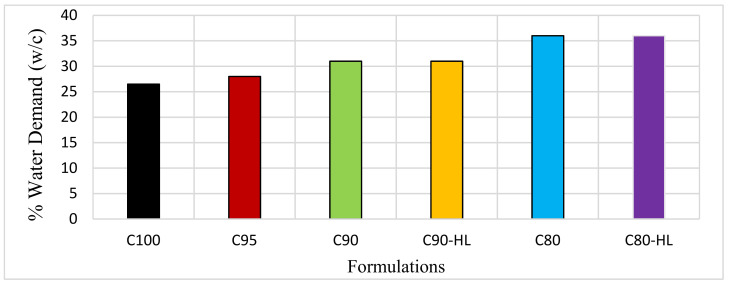
Effect of waste burnt brick powder on water demand of self-compacting paste formulations.

Figure 6 shows that the WD of the paste increased by incorporating WBBP as SCM. The increase in the water demand can be explained based on the WBBP particle size, morphology, and SEM images, as shown in Figure 5a,b. Rizwan and Yijin reported that the water demand of a paste containing SCM not only depends on the cement quantity and fineness but also on the shape, size, and morphology of SCM [18,73]. The WBBP particles are very fine in size and have a large surface area that increases the water demand. Further, SEM images indicate that the particles of WBBP are irregular with a rough surface, which might be a reason for the increased WD of the formulations containing WBBP [74].

#### 3.1.2. Super Plasticizer (SP) Demand

**Figure 7:**
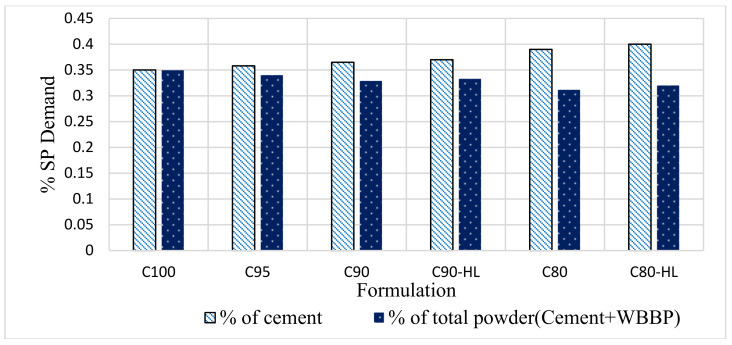
Superplasticizer demand of self-compacting paste formulations.

In Figure 7, the SP demand can be seen as a ratio of the weight of cement and total powder content. The SP demand was reduced as the WBBP replacement percentage was increased because of a reduction in the quantity of the cement. However, an increase in the SP demand was noted in C90-HL and C80-HL as compared to C90 and C80, respectively. Both C90 and C90-HL had a constant water/cement ratio. However, C90-HL had an additional ingredient, HL, as a chemical additive. Therefore, C90-HL required a higher dosage of SP than C-90 for achieving the average target flow of 30 ± 1 cm. The same applies to the reason for the increased SP dosage in C80-HL compared to C80.

#### 3.1.3. Flow Time

**Figure 8:**
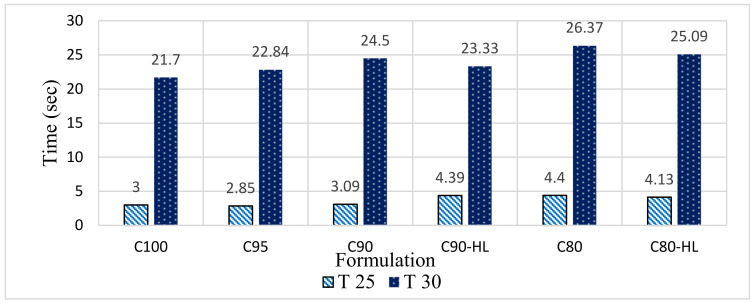
Effect of waste burnt brick powder on T25 cm (paste yield strength parameter) and T30 cm (paste viscosity parameter) of self-compacting paste formulations flow.

The flow time of all six formulations was studied at their respective WD and SP demands. The T25 cm and T30 cm times of the control formulation (Figure 8) are less than the WBBP formulations. This is because cement particles are spherical [75] and thus they offer little resistance to deformation, while WBBP particles are irregular in shape (Figure 5) and thus offer higher resistance to deformation compared to cement particles. The higher values of the T25 cm and T30 cm times of WBBP formulations than C100 indicate a high yield strength and viscosity [60]. Thus, SCP having WBBP as a cement replacement will offer better resistance to segregation and bleeding due to the high yield strength and viscosity values [76,77].

#### 3.1.4. Setting Time

**Figure 9:**
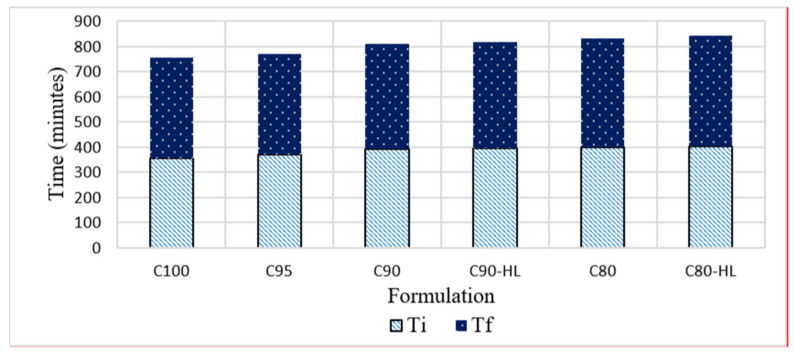
Effect of waste burnt brick powder on setting time (Ti = initial set, Tf = final set) of self-compacting paste formulations.

The setting time of paste depends on the particle size and tri-calcium silicate (C_3_S) content of the cement [78,79]. The cement paste sets quickly if it contains a high content of the C_3_S phase. C_3_S is mainly responsible for the initial setting time (Ti). Figure 9 shows that the control formulation achieved Ti faster than formulations containing WBBP because of the higher amount of C_3_S in the control mix. However, the final setting time (Tf) of the formulations does not show much variation because the activation energy remains constant at the final setting time for natural pozzolan replacements up to 20% [80].

#### 3.1.5. Early-Age Shrinkage

**Figure 10:**
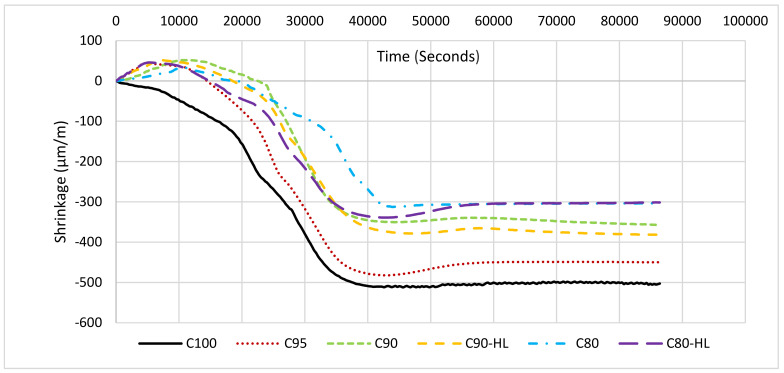
Self-compacting paste formulation shrinkage at the various percentages of waste burnt brick replacement.

It is shown in Figure 10 that the addition of WBBP as SCM caused a reduction in overall shrinkage, which can be supported by the literature where the addition of SCM in SCC caused a significant reduction in volumetric changes in the pastes [18,60,81]. At 20% replacement with WBBP, the shrinkage value reduced to −300 from −500 µm/m. A small expansion was noted at the early hours in formulations having WBBP. According to Rizwan, this small expansion occurred due to the growth of expansive species such as calcium hydroxide and ettringite, and the thermal gradient, and may also be due to the absorption of bleed water in the system [60,82,83]. However, a small increase in shrinkage values was observed in formulations having HL as a modifier, which was probably due to the increased SP demand. The addition of SP increased shrinkage because of the de-flocculation of cement particles [84]. Overall, the WBBP addition as SCM improved the volumetric stability of SCP.

### 3.2. Hardened State Properties

#### 3.2.1. Mechanical Strength

**Figure 11:**
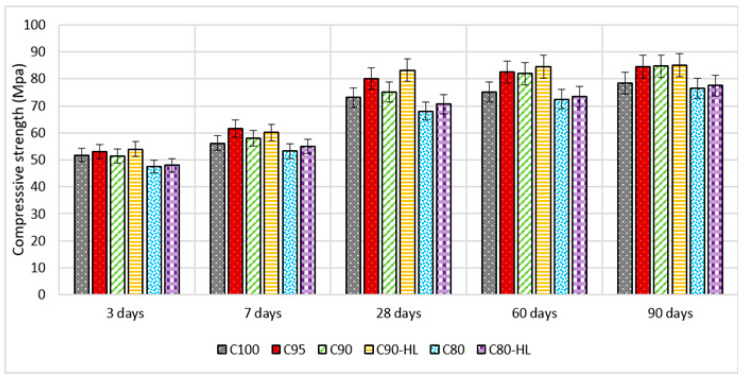
Self-compacting paste formulations’ compressive strength at various replacement percentages.

**Figure 12:**
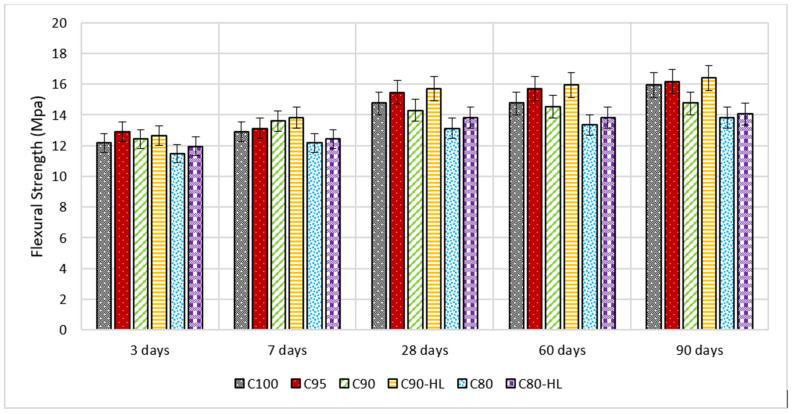
Self-compacting paste formulations’ flexural strength at various replacement percentages.

**Figure 13:**
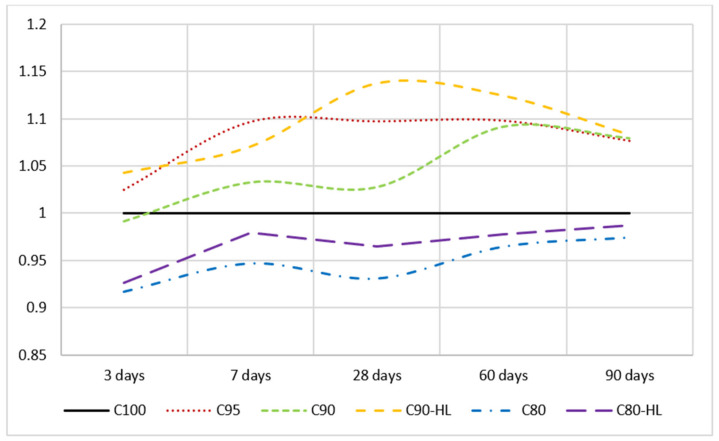
Relative strength index of self-compacting paste formulations at various waste burnt brick powder contents.

Flexural and compressive strengths at the ages of 3, 7, 28, 60, and 90 days are shown in Figure 11 and Figure 12, respectively. The results indicate that the compressive and flexural strengths of C95, C90, and C90-HL were higher as compared to C100 at 3, 7, 28, 60, and 90 days. However, the strength-gaining capability of C80 was comparatively low until 28 days. However, after 28 days, the strength index became higher than 0.95, as shown in Figure 13, and at the age of 90 days, the strength became almost equal to C100. It is worth noting that WBBP formulations having HL (C90-HL and C80-HL) showed improved mechanical properties compared to formulations without HL (C90 and C80). Further, it can be seen in Figure 11 and Figure 12 that C90-HL showed superior mechanical properties compared to the other formulations.

The enhancement in the strength of specimens containing WBBP up to 10% and HL can be attributed to the pozzolanic reaction [54], better pore refinement [18], and filler effects [85]. However, the reduced early-age strength of C80 was due to the high replacement percentage of cement with WBBP [86,87]. The high percentage of cement replacement with pozzolans slows down the pozzolanic action due to the unavailability of sufficient calcium hydroxide at the early age of hydration of cement [66]. The addition of HL improved the early strength, but it does not have a telling effect on the long-term strength, which might be due to the presence of sufficient portlandite from the hydration of cement at later stages.

Moreover, the degree of pozzolanic activity of any SCM depends on its contribution to the strength. The higher the contribution ability of SCM in strength, the greater the pozzolanic potential [88]. Previous researchers’ works suggested that WBBP is a good SCM due to its pozzolanic properties [86,87,89]. It can also be seen in Figure 13 that the relative strength index of WBBP formulations at all ages is greater than 0.9. This indicates that WBBP has very high pozzolanic potential and can be used as a sustainable construction material.

#### 3.2.2. Water Absorption

**Figure 14:**
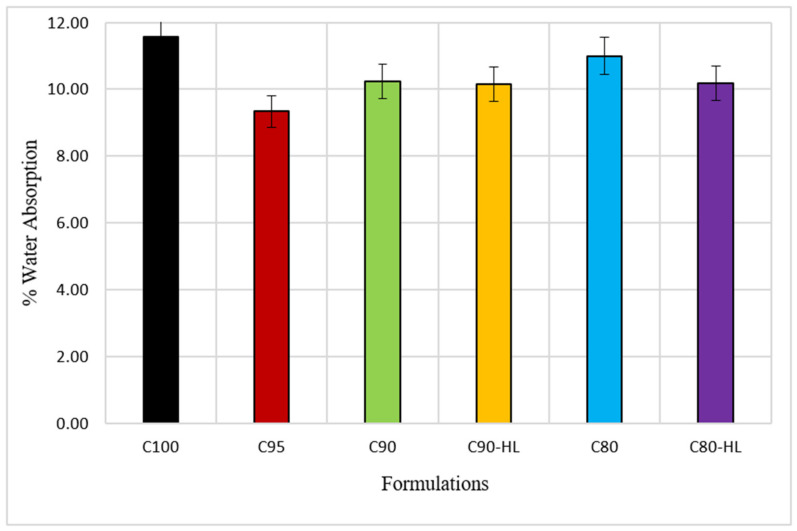
Effect of waste burnt brick powder on water absorption of self-compacting paste formulations.

The results in Figure 14 indicate that in comparison to C100, specimens containing WBBP show a decrease in water absorption. This decrease in water absorption of the paste systems depicts a reduction in porosity. Therefore, paste systems containing WBBP are less porous and will be more durable than C100. However, the WBBP formulations without HL show an increase in water absorption as compared to WBBP formulations with HL. This shows that the HL addition reduced the porosity of the overall system. Therefore, it is sagacious enough to state that replacement specimens have a more compact and denser microstructure compared to C100.

#### 3.2.3. Resistance against Acid Attack

**Figure 15:**
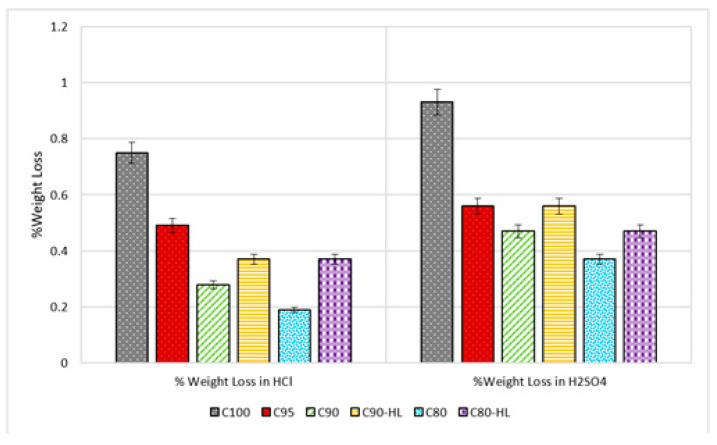
Percentage weight loss of samples exposed to the acidic environment.

**Figure 16:**
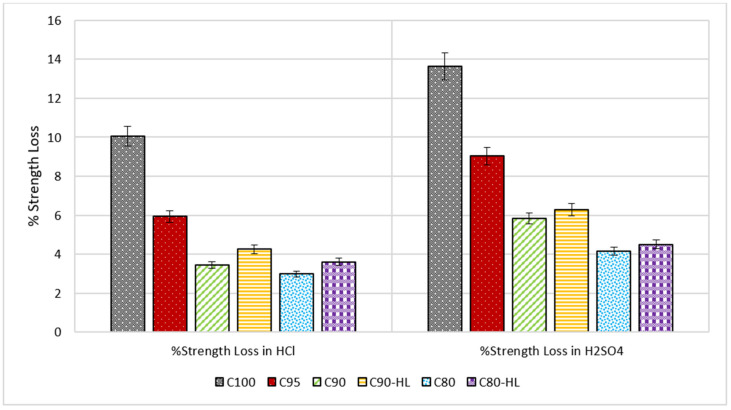
Percentage loss in compressive strength after exposure to the acidic environment.

It can be observed from Figure 15 and Figure 16 that a reduction in the weight and strength of SCP formulations occurred when exposed to the acidic environment. However, the performance of C100 as compared to the remaining five formulations in both acidic environments was very poor. This was because C100 contained a high content of free calcium hydroxide that reacted with the acid to form a slushy mass (gypsum). That slushy mass percolated out of the paste [14]. Meanwhile, the formulations containing WBBP showed better performance because of the presence of the reduced amount of free calcium hydroxide content and secondary CSH gel that formed as a result of a pozzolanic reaction [66,90]. The greater downturn in weight caused by sulfuric acid than HCl was because of the formation of calcium-sulfoaluminate-hydrate, also known as ettringite [Ca_6_Al_2_(SO_4_)_3_(OH)_12_·26H_2_O] [90]. This delay formed ettringite expansions, hence disrupting the set cement paste, whereas no such product was formed in the case of hydrochloric acid [66]. Another reason for the greater weight reduction in the sulfuric acid solution than the HCL solution was the huge quantity of gypsum production due to the high concentration of sulfate ions [91,92,93,94,95]. SCP formulations containing WBBP showed better strength performance in both acidic solutions as compared to the control mix (C100). This might be the reason that pozzolans effectively utilized the free lime and formed a dense matrix accompanied by reduced porosity [14]. It is therefore intuited, based on the test results, that SCP formulations containing WBBP can be used in acidic environments such as chemical industries, sewer pipes, and coastal structures.

### 3.3. Microstructure Investigations

#### 3.3.1. Scanning Electron Microscopy (SEM)

**Figure 17:**
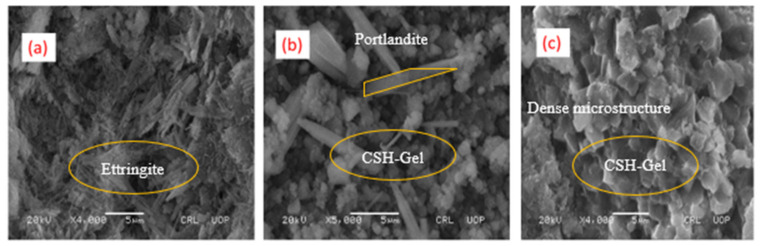
Scanning electron microscopy (SEM) images of self-compacting pastes at 28 days: (**a**) C10; (**b**) C90; (**c**) C90-HL.

Micrographs were taken for some selected formulations (C100, C90, and C90-HL) at the age of 28 days using a scanning electron microscope, as shown in Figure 17. The micrographs of each SCP formulation show the growth of ettringite in the form of needle-shaped crystals, hexagonal prismatic crystals of calcium hydroxide, and amorphous calcium silicate hydrate (C–S–H). The microstructures of C90 and C90-HL are denser and more compact as compared to C100. This is due to the pozzolanic reaction between the active silica and alumina present in WBBP and lime liberating in the cement hydration to form CSH and CAH hydrates, which filled open pores to form a compact structure. As a result, an improvement in the strength occurred. The high strength and better performance against the acid attack of SCP formulations containing WBBP are reflected in SEM images in the form of dense microstructures.

#### 3.3.2. X-ray Diffraction (XRD)

**Figure 18:**
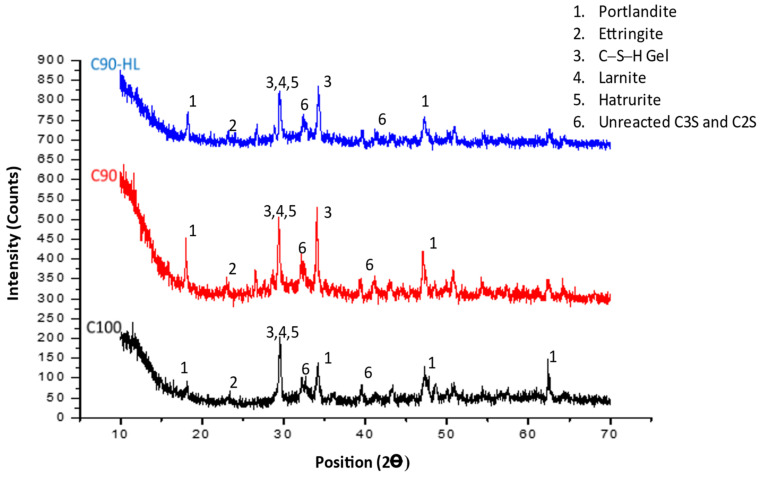
X-ray diffraction of self-compacting paste formulations at 28 days.

XRD is widely used to identify various crystals present in cementitious systems. Portlandite, calcium silicate hydrate (C–S–H) gel, and ettringite are the common hydration products that can be detected by the XRD technique at different 2Ө angles due to their crystalline nature. For some selected formulations (C100, C90, and C90-HL) at the age of 28 days, XRD analysis was carried out at the 2Ө range from 10° to 70° to spot alterations in the hydration products. XRD patterns of the selected SCP formulations are shown in Figure 18. The reduced number of diffraction peaks of portlandite in C90 and C90-HL reflects the potential of WBBP as a pozzolanic material. The reduction in portlandite is also reflected in the form of the better performance of these formulations in terms of strength, water absorption, and resistance against the acid attack. Ettringite [Ca_6_Al_2_(SO_4_)_3_(OH)_12_⋅26(H_2_O)], hatrurite [Ca_3_SiO_5_], larnite [Ca_2_(SiO_4_)], and unreacted calcium silicate diffraction peaks can be seen in all three formulations. The diffraction peak of CSH after 34 2Ө angles in C90 and C90-HL indicates the formation of a secondary CSH gel as a result of the pozzolanic reaction. However, no such prominent CSH peak after 34 2Ө can be found in C100. 

## 4. Conclusions

This research work evaluated the potential of solid waste bricks as a pozzolanic material and hydrated lime as a chemical additive to enhance the pozzolanic reactivity of burnt brick powder. Detailed experimental investigations of the self-compacting paste (SCP) of six formulations were carried out. Based on the current study, the following conclusions can be drawn.

1. The material characterization of waste burnt brick powder (WBBP) through XRD and XRF shows that it is rich in silica and alumina. Further, the summation of its SiO_2_, Al_2_O_3,_ and Fe_2_O_3_ oxides is greater than 75%, which indicates its pozzolanic nature.
2. The water demand (WD) of WBBP formulations is higher than the control mix (C100). The increase in WD for WBBP is due to the large surface area arising from the smaller particle sizes. The particles of WBBP are irregular in shape, having a rough texture and porous surface that also cause an increase in WD.
3. The incorporation of waste burnt brick powder decreases the superplasticizer demand due to the overall reduction in the cement quantity. The rheological properties of the WBBP paste system improved, accompanied by reduced early linear shrinkage as compared to the control mix.
4. Using WBBP as SCM causes a delay in the initial setting time of pastes as compared to the control mix, but the variation in the final setting time is indiscernible.
5. The mechanical and durability performances of WBBP mixes improved due to the consumption of free lime in the pozzolanic reaction. The 10% replacement of cement with WBBP showed better mechanical performance than the reference SCP formulation at 3, 7, 28, 60, and 90 days. The long-term strength (60 and 90 days) of 20% replacement formulations of WBBP was equal to 98% of the control mix. The better durability performance of WBBP mixes than the control mix is due to a more compact and denser matrix structure.
6. Hydrated lime (HL) acts as a triggering agent to enhance the early-age strength due to activation of the pozzolanic reaction of WBBP with HL. However, it has no significant impact on the long-term strength because at later stages of cement hydration, sufficient calcium hydroxide is available in the cement matrix for the pozzolanic reaction.
7. Using waste burnt brick powder as a cement replacement will produce cost-effective, sustainable, and durable self-compacting concrete. Moreover, utilization of WBBP in concrete will reduce solid waste which has considerable positive environmental impacts.

It is recommended that further research should be carried out on WBBP in self-compacting paste systems using higher than 20% replacements partnered with different contents of HL. Further, detailed studies should be carried out on self-compacting concrete using WBBP as a binary or tertiary mineral additive with other secondary raw materials such as fly ash, blast furnace slag, and silica fumes. Moreover, several applications of self-compacting concrete need to be explored such as double-skin tubular members and other components of tall buildings [96,97,98].

## Figures and Tables

**Table 1:** Self-compacting paste formulations.

Serial	Formulation	Cement (%)	W/C (%)	SP (%)	WBBP (%)	HL (%)
1.	C100	100	26.5	0.35	0	0
2.	C95	95	28	0.358	5	0
3.	C90	90	31	0.365	10	0
4.	C90-HL	90	31	0.37	10	2.5
5.	C80	80	36	0.39	20	0
6.	C80-HL	80	36	0.4	20	2.5

Note: W/C = water/cement ratio, SP = superplasticizer, WBBP = waste burnt brick powder, HL = hydrated lime.

**Table 2:** Chemical composition of cement, waste burnt brick powder (WBBP), and hydrated lime (HL).

Sr.No	Chemical Composition	Cement (% by Weight)	WBBP (% by Weight)	HL (% by Weight)
1.	SiO_2_	19.19	69.85	–
2.	Al_2_O_3_	4.97	5.83	–
3.	Fe_2_O_3_	3.27	4.43	4.23
4.	CaO	61.8	15.67	92.53
5.	K_2_O	0.51	1.04	–
6.	MnO	2	0.18	–
7.	ZnO	0.68	0.30	–
8.	SrO	0.29	0.62	3.24
9.	SO_3_	–	0.23	–
10.	MgO	2.23	1.04	–
11.	Na_2_O	0.57	0.81	–
12.	LOI	3.01	4.3	–

**Table 3:** Physical properties of cement, waste burnt brick powder (WBBP), and hydrated lime (HL).

Serial	Physical Properties	Cement	WBBP	HL
1.	Specific gravity	3.15	2.28	2.34
2.	Blain fineness	3100	–	–
3.	D_50_ (µm)	6.85	2.76	10.47
4.	Mean average size	8.44	3.17	10.94
5.	Normal consistency (%)	26.6	–	–
6.	Initial setting time (min)	133	–	–
7.	Final setting time (min)	177	–	–
8.	Soundness value (mm)	3	–	–
9.	Color	Gray	Reddish Brown	White

**Table 4:** Mixing water properties’ comparisons with World Health Organization (WHO) guidelines for potable drinking water.

Sr.No	Parameters	Units	Sample Results	WHO Guidelines
1.	pH	–	6.9	6.5–8.5
2.	Turbidity	NTU	0.7	<0.5
3.	TDS	mg/L	460	<500
4.	Chlorides	mg/L	78	<250
5.	Hardness	mg/L	330	<500

**Table 5:** Properties of Viscocrete 3110.

Serial	Parameter	Property
1.	Form	Liquid
2.	Color	Colorless to yellowish
3.	Water reduction	30%
4.	Shelf life	1 year
5.	Density	1.085 kg/L
6.	Max. dosage	1.7%

## Data Availability

The data used in this study is available on request from the corresponding author.
